# TRAIP modulates the IGFBP3/AKT pathway to enhance the invasion and proliferation of osteosarcoma by promoting KANK1 degradation

**DOI:** 10.1038/s41419-021-04057-0

**Published:** 2021-08-04

**Authors:** Mi Li, Wei Wu, Sisi Deng, Zengwu Shao, Xin Jin

**Affiliations:** 1 Department of Orthopedics, Tongji Hospital, Tongji Medical College, Huazhong University of Science and Technology, Wuhan, China; 2 Department of Orthopedics, Union Hospital, Tongji Medical College, Huazhong University of Science and Technology, Wuhan, China; 3 Cancer center, Union Hospital, Tongji Medical College, Huazhong University of Science and Technology, Wuhan, China; 4 Department of Urology, The Second Xiangya Hospital, Central South University, Changsha, Hunan China; 5 Uro-Oncology Institute of Central South University, Changsha, Hunan China

**Keywords:** Bone cancer, Oncogenes

## Abstract

Osteosarcoma is one of the most common primary malignancies in bones and is characterized by high metastatic rates. Circulating tumor cells (CTCs) derived from solid tumors can give rise to metastatic lesions, increasing the risk of death in patients with cancer. Here, we used bioinformatics tools to compare the gene expression between CTCs and metastatic lesions in osteosarcoma to identify novel molecular mechanisms underlying osteosarcoma metastasis. We identified TRAIP as a key differentially expressed gene with prognostic significance in osteosarcoma. We demonstrated that TRAIP regulated the proliferation and invasion of osteosarcoma cells. In addition, we found that TRAIP promoted KANK1 polyubiquitination and subsequent degradation, downregulating IGFBP3 and activating the AKT pathway in osteosarcoma cells. These results support the critical role of the TRAIP/KANK1/IGFBP3/AKT signaling axis in osteosarcoma progression and suggest that TRAIP may represent a promising therapeutic target for osteosarcoma.

## Introduction

Mesenchymal stem cell-derived osteosarcoma is one of the most common primary malignancies in bones [1], and it is particularly common in children and adolescents. With the recent progress in surgical oncology and neoadjuvant chemotherapies, the 5-year survival rate of patients with osteosarcoma has increased from approximately 20–70% [2]. Nearly one-fifth of osteosarcomas are diagnosed at a metastatic stage [3], and the survival rate of patients with metastatic osteosarcoma is less than 20% [4] due to the lack of effective treatments for these patients.

Circulating tumor cells (CTC) are cancer cells from solid tumors or metastatic lesions that circulate in the body through the peripheral blood [5]. Although most CTCs undergo apoptosis or phagocytosis, some survive and give rise to metastatic lesions, increasing the risk of death in patients with cancer [6]. Recent developments in bioinformatics tools have played an important role in elucidating the molecular mechanisms underlying cancer development and progression. However, the use of in silico tools to better understand CTCs remains limited.

Tumor necrosis factor (TNF) receptor-associated factor (TRAF)-interacting protein (TRAIP) is a RING domain-containing protein acting as an E3 ligase [7]. TRAIP has been implicated in the development of various cancer types [8, 9]. Nevertheless, the role of TRAIP in osteosarcoma remains unknown. In this study, we used bioinformatics to compare the gene profiles between CTCs and metastatic lesions in osteosarcoma to uncover the molecular mechanisms underlying osteosarcoma metastasis. We identified TRAIP as a differentially expressed gene (DEG) with prognostic and diagnostic significance. We also found that TRAIP regulated the proliferation and invasion of osteosarcoma cells. Notably, TRAIP activated the AKT pathway by promoting KANK1 polyubiquitination and degradation and downregulating IGFBP3 in osteosarcoma cells. These findings suggest that TRAIP may represent a promising candidate for osteosarcoma targeted therapy.

## Materials and methods

### Identification of DEGs between CTCs and metastatic lesions

A publicly available gene expression dataset, GSE140131 [10], was downloaded from the Gene Expression Omnibus (GEO) repository (http://www.ncbi.nlm.nih.gov/geo) [11]. The dataset included gene expression data from five CTC samples and three metastatic tumor samples. Expression data were analyzed using R version 4.04 and Bioconductor, and DEGs were identified using the LIMMA package. Genes with FDR values < 0.05 and absolute fold change (FC) > 2 were considered DEGs. The online tool iDEP (http://bioinformatics.sdstate.edu/idep/) [12] was used to constructed an interactive map illustrating the DEGs on the genome. PREDA [13] was used to identify genomic regions significantly enriched with upregulated or downregulated genes.

### Construction of protein-protein interaction networks

The STRING database (http://string-db.org) [14] was used to construct a protein-protein interaction (PPI) network based on the DEGs; a medium interaction confidence score (> 0.4) was used. The network was visualized using Cytoscape version 3.8.2 [15], and the hub genes were identified using MCODE [16] with the following criteria: MCODE score > 5.5, degree cutoff = 2, score cutoff = 0.2, max depth = 100, and k-score = 2.

### Gene ontology and KEGG pathway enrichment of hub genes

Gene ontology (GO) and KEGG pathway enrichment analyses of hub genes were conducted using WebGestalt (http://www.webgestalt.org) [17]. Overrepresentation analysis (ORA) was selected as the method of interest; biological process (BP), cellular component (CC), and molecular function (MF) GO terms were evaluated. KEGG pathway enrichment analysis was also performed. The terms of hub genes were carried out separately with an indicated statistical significance of *p*-value < 0.05.

### Regulatory network of the hub genes

The online database miRDB (http://mirdb.org/mining.html) [18] was used to identify the microRNAs (miRNAs) predicted to bind to the hub genes (confidence score > 60). The database AnimalTFDB 3.0 (http://www.bioguo.org/AnimalTFDB/) [19] was used to identify the transcription factor (TFs) predicted to regulate the expression of the hub genes (*p*-value < 0.01). TF-hub gene and hub gene-miRNA networks were visualized using Cytoscape.

### Weighted gene co-expression network analysis

Weighted gene co-expression network analysis (WGCNA) [20] of genes in the GSE114237 dataset was performed to identify co-expression gene modules and generate a topologic overlap matrix (TOM); different gene modules were identified according to the degree of dissimilarity. The most significant modules in the module-trait relationship and the common hub were used for further analyses.

### Survival analyses

The online R2 genomics analysis and visualization platform (http://r2.amc.nl) was used to analyze the overall survival and metastasis-free survival of patients based on the expression levels of different genes of interest. According to an optimum survival cutoff established based on statistical testing instead of the average or median, the Kaplan scanner separates the samples of a dataset into two groups based on the gene expression of one gene.

### Cell lines and cell culture

Human osteosarcoma cell lines (MNNG/HOS, MG-63, and U-2OS) were obtained from the Cell Bank of China Academy of Sciences (Shanghai, China). All cells were authenticated via STR profiling. Mycoplasma contamination was regularly tested. All cell lines were maintained in α-MEM (HyClone, USA) supplemented with 10% fetal bovine serum (FBS; Gibco, USA) at 37 °C in a 5% CO_2_ humidified atmosphere.

### Western blotting

Cells were lysed on ice in RIPA buffer (Beyotime Institute of Biotechnology, Guangzhou, China) containing a protease inhibitor cocktail (Beyotime Institute of Biotechnology, Guangzhou, China). Protein concentrations were determined by the bicinchoninic acid (BCA) assay. Proteins were separated by SDS-PAGE and transferred onto polyvinylidene difluoride (PVDF) membranes (Pierce Biotechnology, USA). Membranes were blocked with 5% skim milk in Tris-buffered saline-Twee (TBST) and were incubated with primary antibodies overnight at 4 °C. After three TBST washed, membranes were incubated with an HRP-conjugated secondary antibody (BOSTER, China) at 20 °C for 1 h. Protein signals were developed using an ECL detection reagent (Thermo Fisher Scientific, USA) and the ChemiDoc XRS imaging system (Bio Rad Laboratories, Inc. Hercules, CA, USA). Band intensities were analyzed using the Image Lab software (Bio Rad Laboratories, Inc. Hercules, CA, USA). GAPDH was used as a loading control.

### Immunoprecipitation (IP)

Firstly, we add the osteosarcoma cells to the appropriate amount of IP cell lysis buffer (containing protease inhibitor), lyse on ice or at 4 °C for 30 min, and centrifuge at 12000 g for 30 min to take the supernatant. A small amount of lysate was taken for Western blot analysis, and the remaining lysate was added with 1 μ g of the corresponding antibody and 10–50 μ L protein A/g-beads to the lysate, which was slowly shaken at 4 °C and incubated overnight. After immunoprecipitation reaction, the sample was centrifuged at 3000 g for 5 min at 4 °C and the protein A/g-beads were centrifuged to the bottom of the tube. Then the supernatant was carefully removed, and protein A/g-beads were washed with 1 ml lysis buffer for 3–4 times. Finally, 15 μ l of 2 × SDS buffer was added and boiled in boiling water for 10 min. The target protein was detected using Western blotting.

### Quantitative real-time PCR

Total RNA was extracted from the osteosarcoma cell lines using the TRIzol reagent (Invitrogen, USA). First-strand cDNA was generated using the random hexamer primer provided in the first-strand cDNA synthesis kit (PrimeScript™ RT reagent Kit, Code No. RR037A). Then, quantitative real-time PCR analysis (qRT-PCR) was conducted using a PCR kit (TB Green™ Fast qPCR Mix, Code No. RR430A) according to the manufacturer’s protocols. Specific primers for each gene were designed using the Primerbank database. All the experiments were performed in triplicate and calibrated to GAPDH, and we used the 2-^ΔΔ^Ct method to quantify the fold change.

### Cell invasion assay

The in vitro cell invasion assay was applied by using a Bio-Coat Matrigel invasion chamber (BD Biosciences). Each transwell chamber was coated with 50 μL matrigel (1: 8, CORNING). Cells with 100 μL serum-free RPMI-1640 medium were incubated in the upper chamber for 24 h (lower chamber containing complete medium). Then cells were fixed in methanol for 15 min and then stained with 1% crystal violet for 20 min. Cell images were taken in three fields under the microscope, and the number of cells penetrating the membrane was counted.

### Tissue microarray and immunohistochemistry (IHC)

Tissue microarray (Cat No. L714901, Bioaitech, CN) and IHC were performed to assess the levels of TRAIP (Cat No. 10332-1-AP, Proteintech; 1:1600 dilution) and KANK1 (Cat No. 20547-1-AP, Proteintech; 1:2000 dilution) in osteosarcoma.

### In vivo tumor growth

BALB/c-nu mice (4–5 weeks of age, 18–20 g) were purchased from Vitalriver (Beijing, China). All animal experiments were performed according to the guidelines set forth by the Chinese National Institutes of Health and followed the protocols approved by the Ethical Committee on Animal Experiments at the Huazhong University of Science and Technology in Wuhan, China. Power analysis is used to calculate the sample size required for animal experiments. Nude mice have injected with 5 × 10^6^ MNNG/HOS cells into the right flank. Mice were randomly divided into three groups (*n* = 5 mice per group). The tumor volumes were determined every other day for four weeks using the formula *V* = (length × width^2^)÷2. All animal procedures were performed according to the guidelines of the Ethics Committee of Tongji Medical College, Huazhong University of Science and Technology (Wuhan, China).

### Plasmids and antibodies

We purchased mammalian expression vectors for Flag-TRAIP and HA-KANK1 recombinant proteins from GeneChem (Shanghai, China). The TRAIP antibody (abs106179) was purchased from Absin (China, working dilution 1:1000); The KANK1 antibody (abs113793) was purchased from Absin (China, working dilution 1:1000); GAPDH (#ab9485) was from Abcam (UK, working dilution 1:5000); AKT (#4691 T) was from Cell Signaling Technology (USA, working dilution 1:1000); Phospho-AKT (Ser473) (#4060 S) was from Cell Signaling Technology (USA, working dilution 1:1000); IGFBP3 (#ab193910) was from Abcam (UK, working dilution 1:1000); Flag (#A5712) was from Bimake (China, working dilution 1:2000).

### RNA interference

The shRNAs were procured from Sigma-Aldrich. Lipofectamine 3000 (Invitrogen, USA) and Opti-MEM medium (Invitrogen, USA) were used for the transfection reactions; Lipofectamine 3000 was used to transfect 293 T cells to shRNA plasmids and viral packaging plasmids (pVSV-G and pEXQV). At 24 h after transfection, the medium was replaced with fresh DMEM containing 10% FBS and 1 mM sodium pyruvate, and 48 h post-transfection, the virus culture medium was collected and added to the MNNG/HOS, U-2OS, and MG-63 cells supplemented with 12 μg/mL of polybrene. At 24 h after infection, the infected cells were selected with 10 μg/mL of puromycin. The shRNA sequences are provided in the Supplementary data Table S2.

### Liquid chromatography-tandem mass spectrometry/mass spectrometry analysis

The 293 T cells transfected with a TRAIP-expressing plasmid were used to identify novel TRAIP-binding proteins. TRAIP was immunoprecipitated using an anti-TRAIP antibody and protein A + G agarose (#P2012, Beyotime Institute of Biotechnology, Guangzhou, China) at 4 °C. Liquid chromatography-tandem mass spectrometry/mass spectrometry (LC-MS/MS) analysis was performed using a Thermo Ultimate 3000 liquid-phase column combined with a Q-Exactive Plus high-resolution mass spectrometer (Shanghai Applied Protein Technology). The data were retrieved using the software max quant (v1.6.6) and the algorithm Andromeda, and the data were obtained from the human proteome reference database of UniProt. Proteins and peptides with a false discovery rate (FDR) of 1% were selected.

### RNA sequencing

A total of 1 µg of RNA per sample was used as the starting material for RNA sequencing (RNA-seq). Sequencing libraries were generated using the NEBNext Ultra RNA Library Prep Kit for Illumina (NEB, USA) following the manufacturer’s instructions, and index codes were added to attribute sequences to each sample. Clustering of the samples was performed on the cBot Cluster Generation System using the TruSeq PE Cluster Kit v3-cBot-HS (Illumina) according to the manufacturer’s instructions. After cluster generation, libraries were sequenced on an Illumina Novaseq platform, and 150 bp paired-end reads were generated. FeatureCounts v1.5.0-p3 was used to count the read numbers mapped to each gene. Differential expression analysis (two biological replicates per condition) was performed using the DESeq2 R package (1.16.1), and the clusterProfiler R package was used to test the statistical enrichment of differentially expressed genes (DEGs) in KEGG (Kyoto Encyclopedia of Genes and Genomes) pathways.

### MTS assay

The proliferation ability of MNNG/HOS, U-2OS and MG-63 cells was assessed using (3-(4,5-dimethylthiazol-2-yl)-5-(3-carboxymethoxyphenyl)-2-(4-sulfophenyl)-2H-tetrazolium) (MTS reagent) (Abcam, ab197010, USA). Briefly, 1000 cells were introduced into 96-well plates with 100 μl DMEM containing 10% FBS and treated with serial small molecular inhibitors under different concentration gradients. 20 μl of an MTS reagent was added to each well three hours before the end of the incubation period following the manufacturer’s instructions. The absorbance of each well was detected at 490 nm with a microplate reader.

### Glutathione S-transferase (GST) pull down assay

Cells were lysed with IP buffer (50 mM Tris-HCl, pH 7.4, 150 mM NaCl, 1% Triton X-100, 1% sodium deoxycholate, and 1% protease inhibitor cocktails) on ice for more than 30 min. GST fusion proteins were immobilized on glutathione-Sepharose beads (GE Healthcare Lifesciences). After washing with lysis buffer, the beads were incubated with cell lysates at 4 C overnight. The beads were then washed six times with binding buffer and re-suspended in sample buffer. The bound proteins were subjected to western blotting analysis.

### Statistical analysis

All data were expressed as mean ± standard deviation (SD). The sample size (*n* = 3) is recognized as a minimum number to detect a pre-specified effect size. Statistical significance was determined using the Student’s *t*-test, one-way ANOVA, or two-way ANOVA. Statistical analyses were conducted using GraphPad Prism 5. *P*-values < 0.05 were considered statistically significant.

Other methods were provided in the supplementary methods.

## Results

### Identification of DEGs

**Fig. 1:**
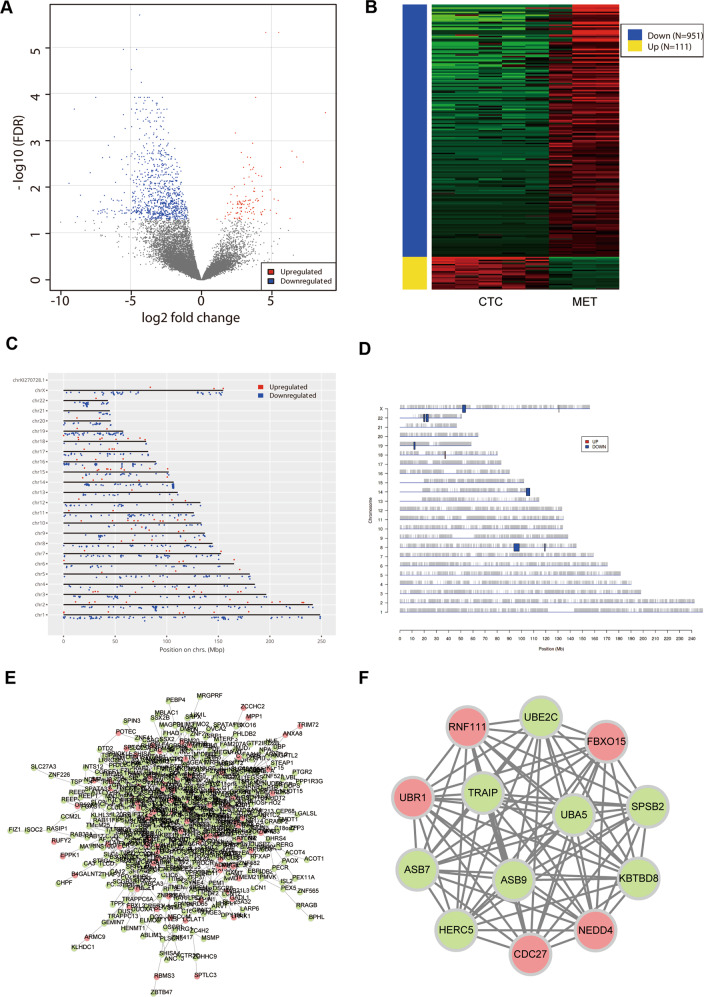
Identification of DEGs. **A** Volcano plot illustrating the DEGs between CTCs and metastatic lesions, with FDR < 0.05 and absolute log fold change (FC) > 1; the red dots represent upregulated genes, the blue dots represent downregulated genes, and the grey dots represent genes with similar expression levels between the groups. **B** Heatmap illustrating the gene expression profiles of CTCs and metastatic lesions. **C** Interactive map showing DEGs on the genome. **D** Significantly enriched genomic regions among the DEGs. **E** The PPI network of the DEGs in Cytoscape. The red dots represent upregulated genes, and the green dots represent downregulated genes. **F** The most significant module in the PPI network. The red dots represent upregulated genes, and the green dots represent downregulated genes.

We identified 1062 DEGs between CTCs and metastatic lesions; 111 genes were upregulated, and 951 were downregulated (Fig. 1A, B). These DEGs were distributed across all chromosomes (Fig. 1C). However, PREDA analysis revealed that most DEGs were on chromosomes 8, 14, 18, 19, 22, and X (Fig. 1D). The DEG-based PPI network was constructed (Fig. 1E), and the most significant module (MCODE score = 13) was identified (Fig. 1F); this module contained 13 nodes and 78 edges. These 13 genes were considered as hub genes and consisted of five upregulated genes and eight downregulated genes, namely ASB7, ASB9, CDC27, FBXO15, HERC5, KBTBD8, NEDD4, RNF111, SPSB2, TRAIP, UBA5, UBE2C, and UBR1.

### Hub genes are associated with protein ubiquitination

**Fig. 2:**
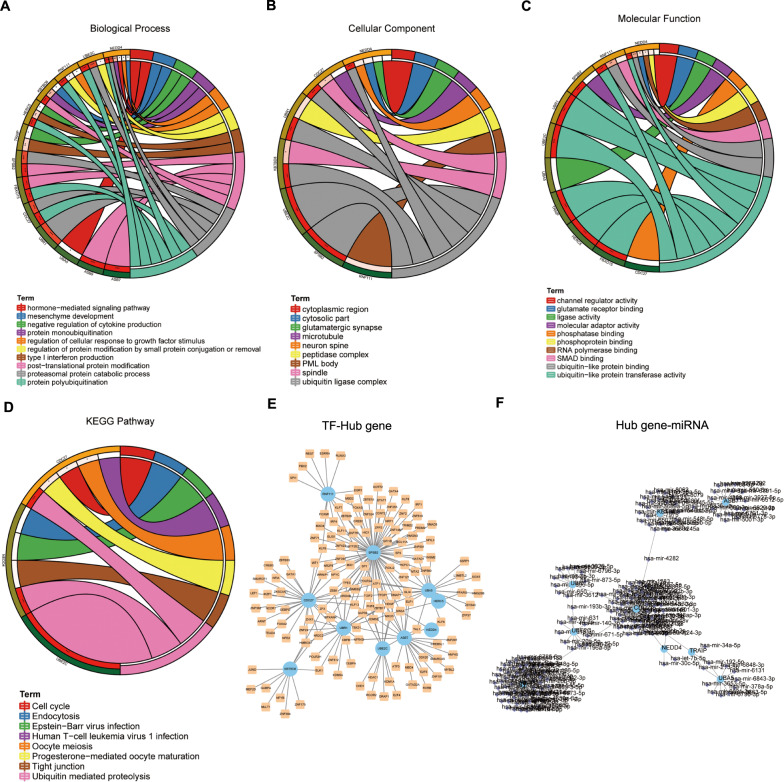
Functional analyses of the hub genes. **A** Enriched biological processes among the hub genes. **B** Enriched cellular components among the hub genes. **C** Enriched molecular functions among the hub genes. **D** Enriched KEGG pathways among the hub genes. **E** The TF-hub gene network in Cytoscape. The blue dots represent hub genes, and the yellow dots represent TFs. **F** The hub gene-miRNA network in Cytoscape. The blue dots represent hub genes, and the purple dots represent miRNAs.

GO and KEGG pathway enrichment analyses of the hub revealed a significant enrichment of ubiquitin-mediated proteolysis (Fig. 2A–D). GO terms associated with protein polyubiquitination, ubiquitin ligase complex, and ubiquitin-like protein transferase activity were also significantly enriched. Additionally, 180 miRNAs (Fig. 2F) and 141 TFs (Fig. 2E) were predicted to regulate the expression of the hub genes.

### WGCNA analysis

**Fig. 3:**
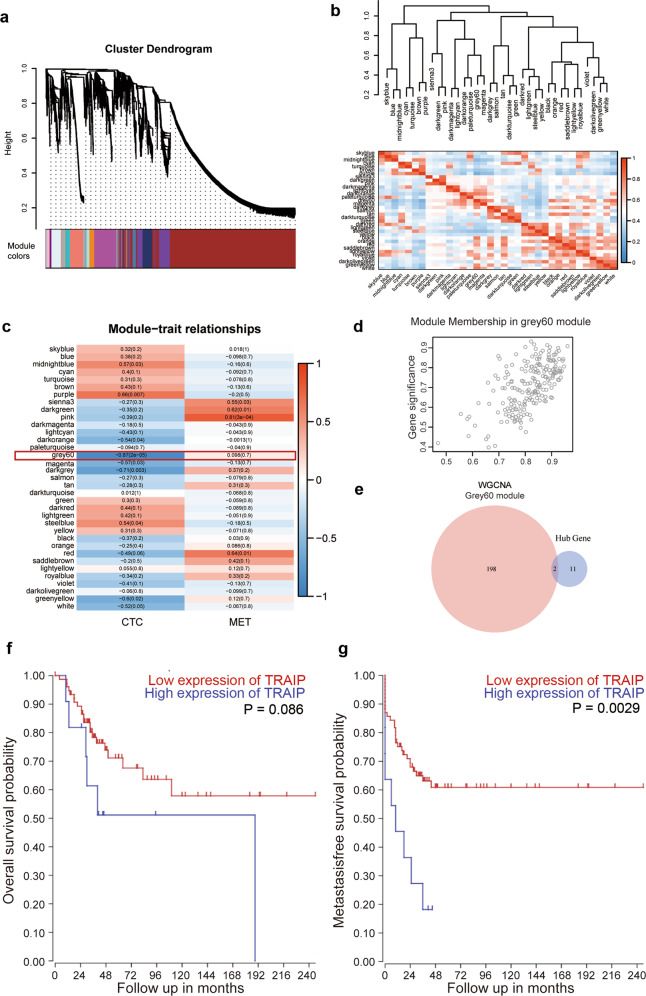
WGCNA analysis of the expression series. **a** Dendrogram of genes based on 1-TOM clustering. **b** Clustering of module eigengenes, showing the correlation between modules. Red indicates a strong correlation, and blue indicates a weak correlation. **c** The correlation between modules and traits. Red indicates a positive correlation, and blue indicates a negative correlation. **d** The relationship between gene significance and module membership of the grey60 module. **e** The intersection of grey60 module-related genes and hub genes. **f** The relationship between TRAIP levels and overall survival in patients with osteosarcoma. **g** The relationship between TRAIP levels and metastasis-free survival in patients with osteosarcoma.

To explore the relationship between CTCs and metastasis in osteosarcoma, we conducted WGCNA and identified more than 30 modules (Fig. 3a, b). The module grey60 (Fig. 3c, d), which contained 200 genes, was the module most positively correlated with CTCs. There were only two common genes identified by differential gene expression analysis and WGCNA (Fig. 3e), namely TRAIP and SPSB2. Considering the FDR and LogFC of TRAIP and SPSB2 between the groups, TRAIP was chosen as our target molecule. Meanwhile, TRAIP was significantly associated with patients’ survival (Fig. 3f, g). This finding suggests that TRAIP may play a role in osteosarcoma progression.

### TRAIP promotes osteosarcoma cell proliferation and invasion

**Fig. 4:**
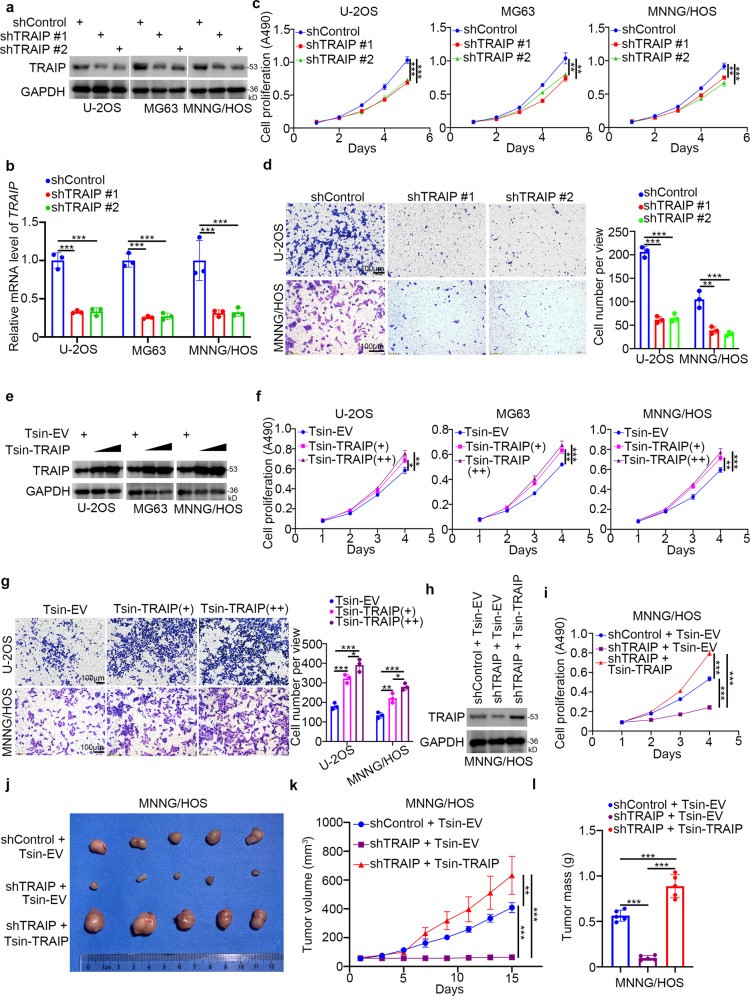
TRAIP promotes osteosarcoma cell growth and invasion. **a**–**d** U-2OS, MG63, and MNNG/HOS cells were infected with indicated lentiviruses expressing shRNAs. After 72 h, cells were used for western blot analysis (**a**), qRT-PCR (**b**), MTS (**c**), and transwell assay (**d**). Data are shown as mean ± SD (*n* = 3). **P < 0.01; ***P < 0.001. **e**–**g** U-2OS, MG63, and MNNG**/**HOS cells were infected with the indicated lentiviruses expressing Tsin plasmids. After 72 h, cells were used for western blot analysis (**e**), MTS assay (**f**), and transwell assay (**g**). Data are shown as mean ± SD (*n* = 3). *P < 0.05; **P < 0.01; ***P < 0.001. **h**, **i** MNNG/HOS cells were infected with the indicated lent**i**viruses expressing shRNAs and Tsin plasmids. After 72 h of puromycine selection, cells were used for western blot analysis (**h**), MTS assay (**i**), and xenograft growth assay in nude mice. **j**–**l** Representative tumor images (**j**), tumor growth curves (**k**), and tumor weights (**l**) are shown. MTS data are shown as mean ± SD (*n* = 3). ***P < 0.001. Xenograft data are shown as mean ± SD (*n* = 5). **P < 0.01; ***P < 0.001.

To determine the role of TRAIP in osteosarcoma, we silenced its expression in osteosarcoma cell lines using two different short-hairpin RNAs (shRNAs) (Fig. 4a, b). *TRAIP* silencing decreased the viability of osteosarcoma cells (Fig. 4c). Furthermore, transwell assay revealed that *TRAIP* silencing suppressed osteosarcoma cell invasion (Fig. 4d). We also stably overexpressed TRAIP in osteosarcoma cell lines using lentiviruses (Fig. 4e). TRAIP overexpression enhanced osteosarcoma cell proliferation and invasion (Fig. 4f, g). To investigate the oncogenic role of TRAIP in vivo, we infected MNNG/HOS cells with lentiviruses expressing control shRNAs, shTRAIP, or shTRAIP plus Tsin-TRAIP (Fig. 4h). Consistently, TRAIP knockdown inhibited MNNG/HOS cell proliferation; rescuing TRAIP expression enhanced cell proliferation (Fig. 4i). These cells were subcutaneously injected into the right flank of nude mice, and tumor growth was followed for four weeks. TRAIP silencing suppressed tumor growth, and rescuing TRAIP expression restored the ability of tumors to grow (Figs. 4j–4l). These data suggest that TRAIP promotes osteosarcoma cell proliferation and invasion.

### KANK1 is a *bona fide* substrate of TRAIP in osteosarcoma cells

**Fig. 5:**
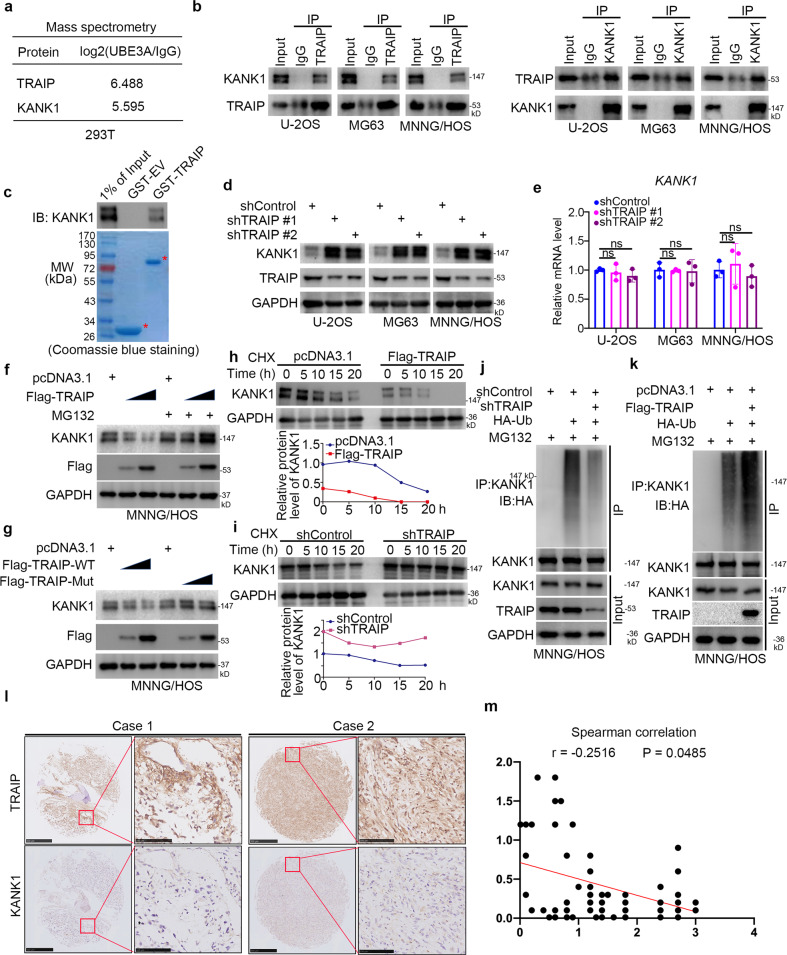
KANK1 is a *bona fide* substrate of TRAIP in osteosarcoma cells. **a** Whole-cell lysates (WCL) of 293 T cells were subjected to mass spectrometry with IgG (control) and anti-TRAIP antibodies. **b** WCL of U-2OS, MG63, and MNNG/HOS cells were subjected to Co-IP with IgG (control) and anti-TRAIP or anti-KANK1 antibodies. **c** Western blot analysis for KANK1 in U-2OS WCL after GST or GST-TRAIP pulldown. The bottom panel shows the Coomassie blue staining of GST or GST-TRAIP protein input. **d**, **e** U-2OS, MG63, and MNNG/HOS cells were infected with the indicated shRNAs. After 72 h, cells were used for western blot analysis (**d**) or qRT-PCR analysis (**e**). Data are shown as mean ± SD with three replicates. ns, not significant. **f** MNNG/HOS cells were transfected with the indicated plasmids for 48 h, and then they were treated with or without 20 µM of MG132 for 8 h. **g** MNNG/HOS cells were transfected with the indicated plasmids for 48 h. Cells were harvested for western blot analysis. **h** MNNG/HOS cells were transfected with the indicated plasmids. After 48 h, cells were treated with cycloheximide (CHX), and cells were collected for western blot analysis at different time points. **i** MNNG/HOS cells were infected with the indicated shRNAs. After 72 h, cells were treated with cycloheximide (CHX), and cells were collected for western blot analysis at different time points. **j** MNNG/HOS cells were infected with the indicated shRNAs. After 72 h, cells were collected for western blotting after treatment with MG132 for 8 h. **k** MNNG/HOS cells were transfected with the indicated plasmids. After 48 h, cells were collected for western blotting after treatment with MG132 for 8 h. **l**, **m** Osteosarcoma tissue microarray was stained for TRAIP and KANK1. Representative images are shown in panel (**l**). The correlation of TRAIP and KANK1 levels is shown in panel (**m**); *P*-values are also shown in the figure.

To explore the mechanisms underlying TRAIP’s ability to promote osteosarcoma progression, we employed mass spectrometry to identify the potential binding partners of TRAIP in 293 T cells (Supplementary Table 3). We identified KANK1 as a TRAIP binding partner (Fig. 5a), which was verified by co-immunoprecipitation in osteosarcoma cells (Fig. 5b) and GST-pull down (Fig. 5c). As TRAIP acts as an E3 ubiquitin ligase [8] and KANK1 has been identified as a tumor suppressor [9], we evaluated whether KANK1 was a substrate of TRAIP. *TRAIP* knockdown resulted in increased KANK1protein levels but did not affect *KANK1* mRNA levels in any of the three osteosarcoma cell lines (Fig. 5d, e). Conversely, TRAIP overexpression decreased KANK1 protein levels; the ability of TRAIP to decrease KANK1 levels was attenuated by the proteasome inhibitor MG132 (Fig. 5f). Consistently, the expression of the TRAIP E3 ligase-dead mutant failed to decrease KANK1 protein levels (Fig. 5g). Moreover, *TRAIP* silencing prolonged the half-life of KANK1 and decreased the ubiquitination levels of KANK1 in MNNG/HOS cells (Figs. 5h, 5i). TRAIP overexpression shortened the half-life of KANK1 and increased the ubiquitination levels of KANK1 in MNNG/HOS cells (Fig. 5j, k). We also investigated the protein of levels of TRAIP and KANK1 in a tissue microarray of osteosarcoma specimens (*n* = 70). We found that there was a relatively weak correlation between TRAIP and KANK1 in osteosarcoma tissues (Spearman *r* = −0.2516, *P* = 0.0485) (Fig. 5l, m). These data suggest that TRAIP functions as an E3 ligase promoting KANK1 degradation in osteosarcoma cells.

### TRAIP enhances osteosarcoma cell proliferation by promoting KANK1 degradation

**Fig. 6:**
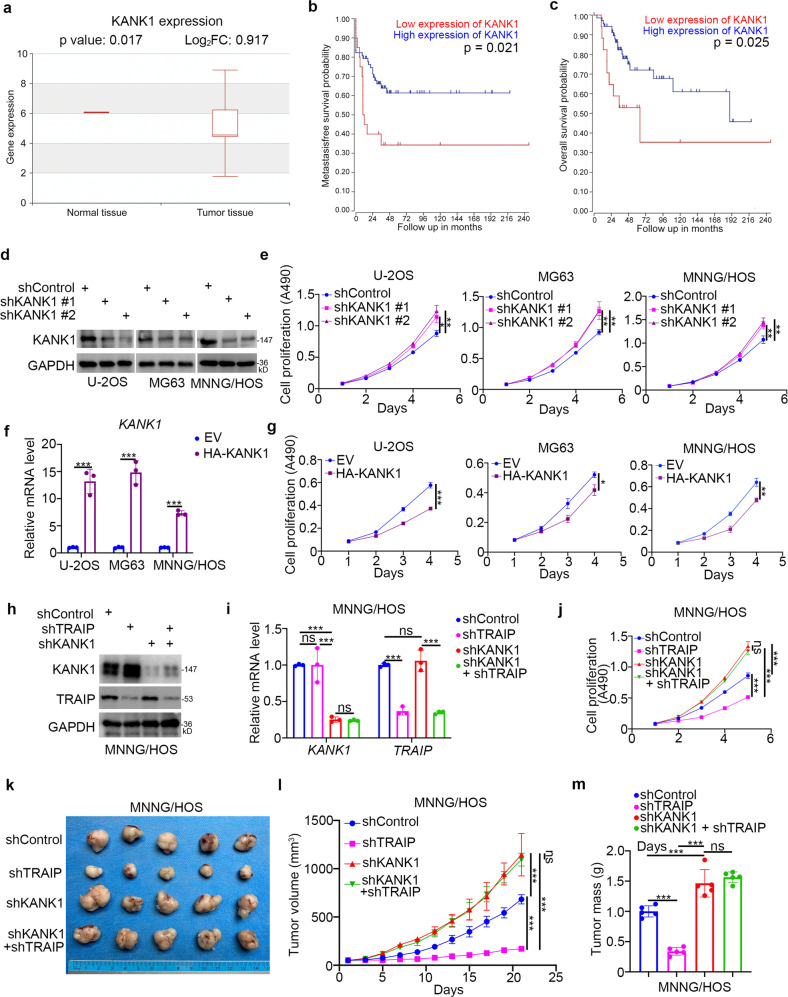
TRAIP enhances osteosarcoma cell proliferation by promoting KANK1 degradation. **a** KANK1 levels in normal tissues and osteosarcoma tissues in the Human Cancer Metastasis Database (http://hcmdb.i-sanger.com/index, HCMDB). **b** Relationship between KANK1 levels and metastasis-free survival in patients with osteosarcoma. **c** Relationship between KANK1 levels and overall survival in patients with osteosarcoma. **d**, **e** U-2OS, MG63, and MNNG/HOS cells were infected with the indicated shRNAs. After 72 h, cells were harvested for western blot analysis (**d**) and MTS assay (**e**). Data are shown as mean ± SD (*n* = 3). *P < 0.05; **P < 0.01. **f**, **g** U-2OS, MG63, and MNNG**/**HOS cells were transfected with the indicated plasmids. After 48 h, cells were harvested for qRT-PCR analysis (**f**) and MTS assay (**g**). Data are shown as mean ± SD (*n* = 3). *P < 0.05; **P < 0.01; ***, *P* < 0.001. (**h–m**) MNNG/HOS cells were infected with the indicated s**h**RNAs. After 72 h, cells were harvested for western blotting (**h**), qRT-PCR analysis (**i**), MTS assay (**j**), and subcutaneous injection into nude mice (**k**–**m**). Representative tumor images are shown in panel (**k**), tumor growth curves are shown in panel (**l**), and tumor weights are shown in panel (**m**). For qRT-PCR analysis and MTS assay, data are shown as mean ± SD (*n* = 3). ***P < 0.001. Xenograft assay data are presented as mean ± SD (*n* = 3). **P < 0.01; ***P < 0.001.

To explore the role of KANK1 in osteosarcoma, we evaluated its expression levels in osteosarcoma tissues. We found that KANK1 was expressed at lower levels in the osteosarcoma tissues than in non-malignant tissues (*P* = 0.017) in The Human Cancer Metastasis Database (http://hcmdb.i-sanger.com/index, HCMDB) (Fig. 6a, Table S3) [21]. Interestingly, high KANK1 levels were associated with prolonged metastasis-free survival (*P* = 0.021) and overall survival (*P* = 0.025) in patients with osteosarcoma (Fig. 6b, c). *KANK1* was knocked down in three osteosarcoma cell lines (Fig. 6d). KANK1 silencing increased osteosarcoma cell proliferation (Fig. 6e), whereas KANK1 overexpression inhibited osteosarcoma cell growth (Fig. 6f, g). These results suggest that KANK1 inhibits the growth of osteosarcoma cells. To evaluate the relevance of KANK1 in TRAIP’s ability to promote osteosarcoma progression, we generated osteosarcoma cells with stable knockdown of *TRAIP*, *KANK1*, and the combination of the two (Figs. 6h, 6i). Intriguingly, co-knockdown of *TRAIP* and *KANK1* diminished the growth inhibition effect of TRAIP knockdown alone in vitro and in vivo (Figs. 6j–6m). These data suggest KANK1 degradation is critical for TRAIP-induced tumor growth in osteosarcoma.

### KANK1 inhibits AKT signaling by upregulating IGFBP3 in osteosarcoma cells

**Fig. 7:**
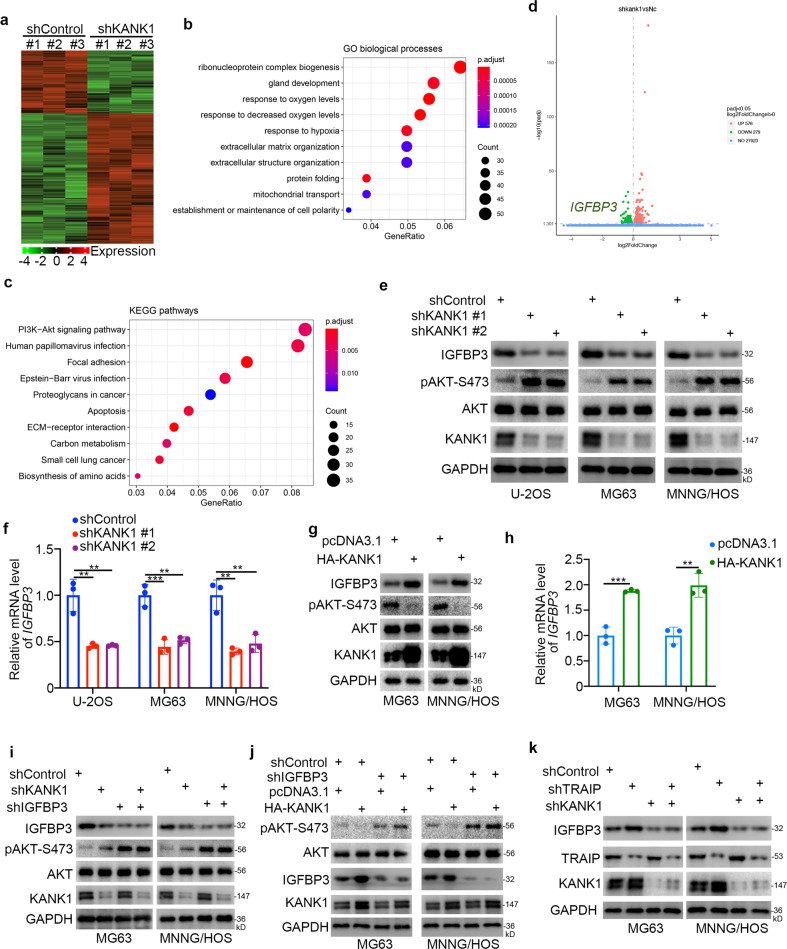
KANK1 inhibits AKT signaling by upregulating IGFBP3 in osteosarcoma cells. **a–d** MNNG/HOS cells were infected with the indicated shRNAs for 72 h. Cells were used for RNA-seq analysis **a**, **d**. GO (**b**) and KEGG pathway enrichment analyses (**c**) were performed. **e**, **f** U-2OS, MG63, and MNNG/HOS cells were infected with the indicated shRNAs. After 72 h, cells were harvested for western blot (**e**) and qRT-PCR analysis (**f**). Data are presented as mean ± SD (*n* = 3). **P < 0.01; ***P < 0.001. **g**, **h** MG63 and MNNG/HOS cells were transfected with the indicated plasmids. After 48 h, cells were harvested for western blotting (**g**) and qRT-PCR analysis (**h**). Data are shown as mean ± SD (*n* = 3). **P < 0.01; ***P < 0.001. **i** Western blot analysis of MG63 and MNNG/HOS cells infected with the indicated shRNAs for 72 h. **j** Western blot analysis of MG63 and MNNG/HOS cells transfected with the indicated constructs for 72 h. **k** Western blot analysis of MG63 and MNNG/HOS cells infected with the indicated shRNAs for 72 h.

To further explore the tumor-suppressive roles of KANK1 in osteosarcoma, we performed RNA sequencing (RNA-seq) analysis after silencing *KANK1* in MNNG/HOS cells (Fig. 7a). GO and KEGG pathway enrichment analysis showed that KANK1 was involved in various cellular processes and pathways (Fig. 7b, c). Notably, the AKT pathway was activated after KAKNK1 silencing (Fig. 7c), and IGFBP3 was one of the most strongly downregulated genes in cells with *KANK1* silencing (Fig. 7d). To confirm the result of RNA-seq analysis, we conducted qRT-PCR in cells with *KANK1* knockdown or overexpression (Fig. 7e–h). *KANK1* silencing decreased *IGFBP3* expression levels and increased AKT phosphorylation levels (Fig. 7e, f). Conversely, KANK1 overexpression upregulated *IGFBP3* and reduced AKT phosphorylation levels in osteosarcoma cells (Fig. 7g, h).

**Fig. 8:**
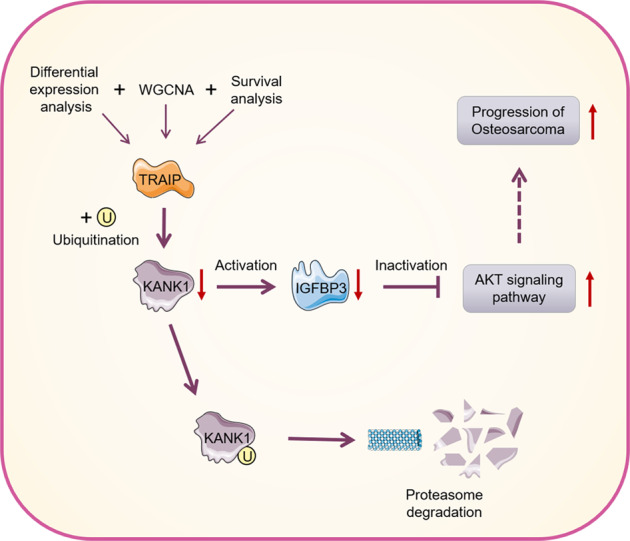
A schematic representation of TRAIP as a key prognostic DEG between CTCs and metastatic lesions in osteosarcoma. TRAIP targets KANK1 for degradation, activating the IGFBP3/AKT signaling pathway and promoting osteosarcoma cell proliferation and invasion.

IGFBP3 negatively regulates the PI3K/AKT signaling pathway [22, 23]. Hence, we next investigated whether IGFBP3 mediated the ability of KANK1 to inhibit the AKT pathway. *IGFBP3* knockdown attenuated the effects of KANK1 silencing or overexpression on AKT phosphorylation levels in MG63 and MNNG/HOS cells (Fig. 7i, j). We also found that TRAIP regulated IGFBP3 expression through KANK1 in osteosarcoma cells (Fig. 7k). These results indicate that the KANK1/IGFBP3 axis modulates the activation of AKT signaling in osteosarcoma cells (Fig. 8).

## Discussion

In this study, we used transcriptomics data to identify the DEGs between CTCs and metastatic lesions in patients with osteosarcoma. DEGs were used to construct a PPI network and identify hub genes. Functional enrichment analyses revealed a significant enrichment of protein ubiquitination-related genes among DEGs. Among these genes, TRAIP displayed a significant prognostic value in osteosarcoma. We confirmed that TRAIP promoted osteosarcoma progression by regulating protein ubiquitination. However, these data are derived from a publicly available dataset with small sample size. Future analyses in large sets of osteosarcoma specimens are required to confirm our results. Besides, we also analyzed the miRNAs and TFs predicted to regulate the expression of the hub genes, such as TRAIP. However, the related experiment is in progress. We are looking forward to uncovering the regulatory mechanism of TRAIP in the near future.

TRAIP has been considered to be an inhibitor of the NK-κB pathway recruited to the TRAF signaling complex by binding to CYLD or Syk [24–26]. Subsequently, TARIP has been shown to ubiquitylate the replicative CMG (CDC45/MCM2–7/GINS) helicase, thereby regulating the NEIL3 pathway and Fanconi anemia pathway in the DNA interstrand crosslink repair process [27]. This TRAIP-dependent CMG regulation has been implicated in replisome disassembly during mitosis [28]. Consistently, TRAIP silencing suppressed G1/S phase transition and inhibited cell proliferation in human epidermal keratinocytes [29]. Moreover, TRAIP silencing in mice was embryonically lethal, suggesting a pivotal role for TRAIP in embryonic development [30]. The role of TRAIP in tumorigenesis has also been reported. TRAIP was found to be overexpressed in liver cancer, and its expression levels were associated with poor prognosis in patients with liver cancer. Moreover, inhibition of TRAIP induced apoptosis in liver cancer cells [8]. Furthermore, the tumor suppressor CYLD [31] is a TRAIP substrate, suggesting that TRAIP may regulate tumorigenesis [25]. In this study, we performed bioinformatics analyses to investigate the role of TRAIP in osteosarcoma. We found that TRAIP levels were associated with metastasis-free survival and overall survival in patients with osteosarcoma. We also found that TRAIP enhanced the malignancy of osteosarcoma cells.

To get further insight into the mechanisms underlying the tumor-promoting roles of TRAIP in osteosarcoma, we performed mass spectrometry and identified KANK1 as a TRAIP substrate. KANK1 belonging to the KANK protein family contains a KANK N-terminal (KN) motif, a coiled-coil domain, and an ankyrin (ANK) repeat domain in the C-terminal [32]. KANK1 plays a critical role in cytoskeletal organization and actin polymerization [32]. The *KANK1* gene is methylated in the kidneys, lungs, and brain tissues, and low KANK1 levels have been associated with tumor progression in various cancer types [33, 34]. It has been documented that KANK1 inhibited cancer development by blocking the Rho-associated protein kinase (ROCK) pathway and the β-catenin pathway [35, 36]. Moreover, KANK1 has been shown to regulate Yap expression, inhibiting cell proliferation and inducing apoptosis in oral squamous cell carcinoma cells [33]. Interestingly, we found that KANK1 was downregulated in osteosarcoma tissues compared with non-malignant tissues. We also found that KANK1 downregulated IGFBP3 in osteosarcoma cells. It has been reported that IGFBP3 could diminished the activation of AKT induced by TGF-β1 in osteosarcoma cells and block cell proliferation and cell cycle progression [37]. Moreover, IGFBP3 was also found to inhibit the osteosarcoma cell migration via attenuating the PI3K/AKT/VCAM-1 signaling [38]. Besides, AKT inactivating the AKT pathway is crucial for tumorigenesis and drug resistance in osteosarcoma [39, 40]. Thus, the KANK1/IGFBP3/AKT pathway is crucial for understanding the specific mechanism of the progression of osteosarcoma.

In conclusion, we used bioinformatics to identify DEGs between CTCs and metastatic lesions in osteosarcoma and identified TRAIP as a key DEG with prognostic significance. Aberrant TRAIP overexpression promoted osteosarcoma cell proliferation and invasion. We also found that TRAIP acted as an E3 ligase, promoting KANK1 degradation, which activated the IGFBP3/AKT signaling pathway (Fig. 8). These findings suggest that TRAIP/KANK1/IGFBP3/AKT signaling axis may represent a promising therapeutic target for osteosarcoma.

## Supplementary information

Supplementary information

Table S3

## Data Availability

The datasets used and/or analyzed during the current study are available from the corresponding authors (Mi Li, ortholimit@hust.edu.cn) on reasonable request.
