# Supplementary material for: TRAIP modulates the IGFBP3/AKT pathway to enhance the invasion and proliferation of osteosarcoma by promoting KANK1 degradation
Source: Cell Death Dis. 2021 Aug 4;12(8):767. doi: 10.1038/s41419-021-04057-0 (PMC8339131; doi:10.1038/s41419-021-04057-0)
Supplement: Supplementary file 1 — Supplementary information [file 41419_2021_4057_MOESM1_ESM.docx]

**TRAIP modulates the IGFBP3/AKT pathway to enhance the invasion and proliferation of osteosarcoma by promoting KANK1 degradation**

Mi Li, Wei Wu, Sisi Deng, Zengwu Shao, Xin Jin

**Table S1: Sequences of RT-qPCR primers**

| **Species** | **Gene** | **Forward (5’-3’)** | **Reverse (5’-3’)** |
| --- | --- | --- | --- |
| Human | *GAPDH* | CCAGAACATCATCCCTGCCT | CCTGCTTCACCACCTTCTTG |
| Human | *TRAIP* | TGGTTGGTGCCTTCCCTATT | ACCCTCACCCTCTGCTTAAC |
| Human | *KANK1* | TCCTGCCAGCCTGAATGTAA | TCTGTGTTGCTGCCTGTTTC |
| Human | *IGFBP3* | TCTGATCCCAAGTTCCACCC | TCCATTTCTCTACGGCAGGG |

**Table S2: Sequences of gene-specific shRNAs**

| shTRAIP-1 | 5′- CCGGGCAGACAGTCTACTCTGAATTCTCGAGAATTCAGAGTAGACTGTCTGCTTTTTG-3′ |
| --- | --- |
| shTRAIP-2 | 5′- CCGGCCCAGCATGGTTACTACGAAACTCGAGTTTCGTAGTAACCATGCTGGGTTTTTG-3′ |
| shKANK1-1 | 5′- CCGGGTATGCAAATAGCCCTTTATTCTCGAGAATAAAGGGCTATTTGCATACTTTTTG-3′ |
| shKANK1-2 | 5′- CCGGCAGAATGGATACCAAGGTAATCTCGAGATTACCTTGGTATCCATTCTGTTTTTTG-3′ |
